# Human Endogenous Retrovirus W Activity in Cartilage of Osteoarthritis Patients

**DOI:** 10.1155/2014/698609

**Published:** 2014-07-22

**Authors:** Signy Bendiksen, Inigo Martinez-Zubiavrra, Conny Tümmler, Gunnar Knutsen, Jan Elvenes, Elisabeth Olsen, Randi Olsen, Ugo Moens

**Affiliations:** ^1^Department of Laboratory Medicine, University Hospital of North Norway, Sykehusveien 38, 9038 Tromsø, Norway; ^2^Department of Clinical Medicine, Faculty of Health Sciences, University of Tromsø, MH-Building, 9037 Tromsø, Norway; ^3^Genøk Centre of Biosafety, Science Park, Sykehusveien 23, 9294 Tromsø, Norway; ^4^Department of Orthopaedics, University Hospital of North Norway, Sykehusveien 38, 3098 Tromsø, Norway; ^5^Department of Electron Microscopy, Institute of Medical Biology, Faculty of Health Sciences, University of Tromsø, Sykehusveien 44, 9037 Tromsø, Norway; ^6^Department of Medical Biology, Faculty of Health Sciences, University of Tromsø, MH-Building, 9037 Tromsø, Norway

## Abstract

The etiology of viruses in osteoarthritis remains controversial because the prevalence of viral nucleic acid sequences in peripheral blood or synovial fluid from osteoarthritis patients and that in healthy control subjects are similar. Until now the presence of virus has not been analyzed in cartilage. We screened cartilage and chondrocytes from advanced and non-/early osteoarthritis patients for parvovirus B19, herpes simplex virus-1, Epstein Barr virus, cytomegalovirus, human herpes virus-6, hepatitis C virus, and human endogenous retroviruses transcripts. Endogenous retroviruses transcripts, but none of the other viruses, were detected in 15 out the 17 patients. Sequencing identified the virus as HERV-WE1 and E2. HERV-W activity was confirmed by high expression levels of syncytin, dsRNA, virus budding, and the presence of virus-like particles in all advanced osteoarthritis cartilages examined. Low levels of HERV-WE1, but not E2 envelope RNA, were observed in 3 out of 8 non-/early osteoarthritis patients, while only 3 out of 7 chondrocytes cultures displayed low levels of syncytin, and just one was positive for virus-like particles. This study demonstrates for the first time activation of HERV-W in cartilage of osteoarthritis patients; however, a causative role for HERV-W in development or deterioration of the disease remains to be proven.

## 1. Background

Osteoarthritis (OA) is one of the most common and painful forms of arthritis striking people worldwide and may affect all articular joints in the human body; however, it is most prevalent in hands, knees, hips, and spines [1]. The disease is commonly defined as a heterogeneous group of conditions that lead to joint pain and malfunction and is characterized by the progressive destruction of articular cartilage in addition to related changes in the subchondral bone and joint margins. Despite being one of the oldest documented diseases (arthritis has been demonstrated in ice-aged skeletons and dinosaur bones [2, 3]), the exact etiology of this disease remains unknown. “Wear and tear” was in earlier days recognized as the main reason for the disease. Today we know that many factors seem to play a causal role in OA, including hereditary predisposition, wrong alignment of limbs, mechanical overloading, chronic inflammation, stress, obesity, ageing, and hormones [4, 5]. Viral infection has also been suggested to play a causative role in the disease, but proof is lacking. Viral genomic sequences of the DNA viruses parvovirus B19, human herpes virus-1 (HHV-1; herpes simplex virus-1), HHV-3 (varicella zoster virus), HHV-4 (Epstein Barr virus), HHV-5 (human cytomegalovirus), transfusion transmission virus, and the RNA viruses GB virus C and endogenous retroviruses have been identified in OA patients [6–20]. However, other groups could not confirm these findings [21–28]. Moreover, viral nucleic acid sequences were detected in peripheral blood mononuclear cells, synovial fluid, or tissue, while cartilage or chondrocytes were not examined. Additionally, these viruses were also detected in non-OA control subjects [6, 8, 9, 11–20]. These findings question the etiological role of viruses in osteoarthritis. To investigate the implication of viral infection in OA, we analyzed cartilage and chondrocytes obtained from advanced OA and non- or early OA patients for signs of active viral infection. For this purpose, we monitored specimens for viral genome sequences, expression of viral proteins, and virus particles. All samples were PCR or reverse-transcriptase PCR negative for parvovirus B19, HHV-1, HHV-3, HHV-5, HHV-6, and hepatitis C virus (HCV) with the exception of human endogenous retrovirus (HERV) which was common in cartilage from advanced OA patients, but not from non-OA and early OA patients. Partial sequencing identified this virus as HERV-W. Viral activity was evident by the presence of dsRNA, viral protein syncytin-1, and virus budding, and virus particles with estimated size corresponding to retroviruses could be visualized in the material of all OA patients tested. With the exception of one, samples of all non-OA and early OA patients did not show signs of viral particles. These findings suggest that activation of the endogenous retrovirus HERV-W is more common in OA patients than in controls, but it remains to be proven if this virus is implicated in the initiation/onset or progression of the disease.

## 2. Methods

### 2.1. Patients

Material from a total of 33 advanced OA patients (age 30–82) and 8 early or non-OA patients (age 25–45) has been included in different parts of these experiments (donor list is summarized in Supplementary Table, in Supplementary Material available online at http://dx.doi.org/10.1155/2014/698609). All participants signed a written informed consent to use biopsies for scientific purposes. The project was approved by the Regional Ethic Committee (REK 61/2007 and 5.2006.161) and experiments were performed in accordance with the Code of Ethics of the World Medical Association (Declaration of Helsinki) for experiments involving humans. The patients involved in this study had incipient to severe osteoarthritis and were classified as grades 0–4 according to the Kellgren-Lawrence (KL) radiological classification of OA. Material analyzed in this study consisted of cartilage tissue and cells derived from patients with advanced osteoarthritis undergoing total joint replacement, and surplus chondrocytes expanded in monolayer cultures from patients with focal cartilage damages, undergoing autologous cell transplantation (autologous chondrocyte implantation (ACI); KL: 0-1). Additionally, healthy cartilage samples used in this study (*n* = 2) were taken from young adults suffering traumatic joint lesion (KL grade 0) in knee.

### 2.2. Cell Cultures

Cartilage biopsies were cut in small pieces and incubated with collagenase type XI and deoxyribonuclease type I and IV from bovine (Sigma-Aldrich, Germany) at 37°C for up to 15 hours. Collagenase-digested tissue was filtrated and spun down to eliminate excess of enzymes. Isolated chondrocytes were washed in PBS and cultured in complete medium, DMEM-F12 (Sigma-Aldrich) supplied with gentamicin (Sigma-Aldrich) and 10% bovine serum (Sigma-Aldrich). Cells from ACI operations were expanded initially in 10% autologous serum to meet the requirements for transplantation in the clinics. Thereafter, surplus cells were transported to the lab and their expansion continued under the abovementioned conditions. Cells were grown to 80% confluence before each subculturing. Chondrocytes from both advanced OA and non-OA were expanded for 3-4 weeks (3 passages) before experimental analyses.

### 2.3. RNA-Isolating and cDNA Synthesis

Medium was removed and cells were washed in PBS; then PBS was removed and the cell pellet was quickly frozen at −2°C, ready for RNA-isolating or resolved in freezing medium (medium with 20% bovine serum and 10% DMSO (Sigma-Aldrich)). Cells were thawed and total RNA was prepared using Qiagen RNeasy Mini Kit (cat. number 74106). The RNA concentrations were measured by NanoDrop ND 1000 Spectrophotometer (Thermo Scientific, Wilmington, DE, USA). Then cDNA was synthesized using Oligo(dT) Primers (Amersham Pharmacia Biotec Inc.) and MonsterScript reverse transcriptase (Epicentre Biotechnologies, Madison, WI, USA) according to the manufactures' protocol.

### 2.4. Polymerase Chain Reaction (PCR)

PCR was performed on cDNA using Phusion High Fidelity DNA polymerase (Finnzymes) and specific primers (Medprobe), listed in Table 1. All PCRs were performed on a PTC-200 Peltier Thermal Cycler; initiated melting; 5 minute, 96°C, followed by 40 cycles with melting temperature 96°C, annealing temperature 58°C, and extension temperature 72°C, all in 30-second intervals, and followed by final extension for 7 minutes at 72°C. The PCR products were applied to an agarose gel before running electrophoresis. DNA band was visualized by UV light and photographed.

### 2.5. Immunocytochemistry

Cells were grown in medium DMEM-F12 supplied with gentamicin and 10% FBS on camber slides (cat. number 177429, Nunc, Roskilde, Denmark) and starved 24 h in serum-free medium and then washed in cold PBS before incubating for 15 minutes with 0.5% saponin (Sigma-Aldrich; cat. number 47036-50G-F) for permeability and then for 2 h in cold PBS containing 2% sucrose and 4% paraformaldehyde. The cells were washed twice in cold PBS supplied with 1% BSA. Anti-dsRNA-specific J2-IgG2-A monoclonal antibody (English & Scientific Consulting, Hungary) was diluted 1 : 100 in PBS added and incubated over night at 4°C. Negative controls were performed by omitting primary antibody in this procedure. The cells were rinsed in PBS before adding Alexa Fluor stained IgG F(ab)2 fragment (Invitrogen) diluted 1 : 400 in PBS supplied with 0.5% BSA as secondary antibody and incubated over night at 4°C or for 2 hours at room temperature in dark. Labeled cell cultures were examined by fluorescence microscopy.

### 2.6. Transmission Electron Microscopy (TEM)

Biopsies from patients were cut in small pieces (1-2 mm^3^) and fixed in McDowell's fixative as previously described [29]. Supernatants collected from collagenase XI-treated cartilages for breaking down matrix proteins were ultracentrifuged for 2 hours to isolate chondrocytes. The small pellets were resuspended in 100 *µ*L PBS and subjected to standard procedures for negative staining by uranyl acetate and microscopy.

### 2.7. Immune Electron Microscopy (IEM)

Cartilage biopsies were cut in small pieces and fixed in 8% formaldehyde in PBS according to standard procedures [30, 31]. The presence of double-stranded RNA (dsRNA) was demonstrated using mouse anti-dsRNA J2-IgG2A monoclonal antibody (English & Scientific Consulting, Hungary), diluted 1 : 50 in PBS supplemented with 1% cold water fish skin gelatin (G-7765; Sigma-Aldrich, St. Louis, MO) to block for nonspecific binding of antibodies. A second rabbit anti-mouse IgG antibody (ICN/Chappel, Aurora, OH) was added, and binding was visualized by protein-A gold (University of Utrecht, The Netherlands). Between each step the specimens were washed in PBS supplied with cold fish skin gelatin. Finally, the grids were washed in distilled water and dried in 1.8% methylcellulose and 0.3% uranyl acetate and examined in a Jeol 1010 Transmission Electron Microscope (Tokyo, Japan).

## 3. Results

### 3.1. Identification of Endogenous Retroviral Transcripts in OA Cartilage and Cultured Chondrocytes

**Figure 1:**
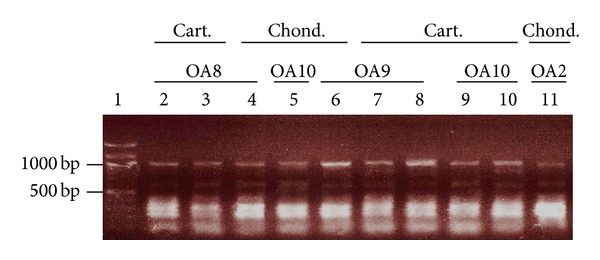
Detection of human endogenous retrovirus sequences in chondrocytes and cartilage from OA patients. Total RNA was isolated and converted into cDNA. HERV sequences were amplified using degenerated primers complementary to sequences in the* pro/pol* genes of HRV-5 (Table 1) to obtain a ~1,000 bp fragment. Lane 1: DNA marker (in kb); lanes 2–4: OA8; lanes 4, 8, and 10: OA10; lanes 6–8: OA9; lane 11: OA2. Lanes 2, 8, and 9: RNA isolated from nitrogen-crushed cartilage; lanes 3, 7, and 10: RNA obtained from enzyme-digested cartilage; lanes 4–6 and 11: RNA purified from culture-expanded chondrocytes. PCR reactions were run on an agarose gel, stained with ethidium bromide, and the DNA was visualized under UV light. Cart.: cartilage; chond.: chondrocytes.

Viral infections, including parvovirus B19, HHV-1, HHV-3, HHV-5, HERVs, and HCV, have been suggested as a causative factor in OA, but solid proof is lacking [6, 8, 9, 11–20]. In fact, studies examining chondrocytes or cartilage of OA patients for the presence of virus are lacking and most studies have only examined the presence of viral genomes, rather than active viral infections. Because several viruses reside in a latent or persistent state in most individuals, the presence of viral mRNA was monitored as a sign of viral activity. Complementary DNA (cDNA) was prepared from RNA isolated from chondrocytes of collagenase-treated cartilage, nitrogen-crushed cartilage, or 3rd passage chondrocyte cultures from OA patients and subjected to PCR using specific primers (Table 1). No PCR products were obtained with primers against parvovirus B19, HHV-1, HHV-4, HHV-5, HHV-6, and HCV (data not shown). In 1999, Griffiths and colleagues detected human retrovirus-5 (HRV-5), a virus later characterized as a rabbit endogenous retrovirus [10, 32]. Using primers complementary to sequences in the* pro/pol* genes of HRV-5, multiple bands of approximately 200, 300, 700, and 1,000 bp were obtained with cartilage tissue and culture-expanded chondrocytes from all OA specimens (*n* = 17). A representative result for OA patients 2, 8, 9, and 10 is shown in Figure 1. The presence of contaminating chromosomal DNA containing integrated endogenous retroviral DNA is unlikely because the isolated RNA was treated with DNase before being converted into complementary DNA (cDNA). Moreover, obtained cDNA was tested with primers against the* adenine phosphoribosyltransferase* (APRT) gene that generate a 300 bp fragment for cDNA and an 800 bp fragment for DNA. Only a 300 bp fragment was obtained (results not shown). These findings underscore that the HERV amplicons were derived from transcripts and not from the chromosomal integrated viral genome.

### 3.2. Virus Identification: Sequencing of the Viral PCR Products

**Figure 2:**
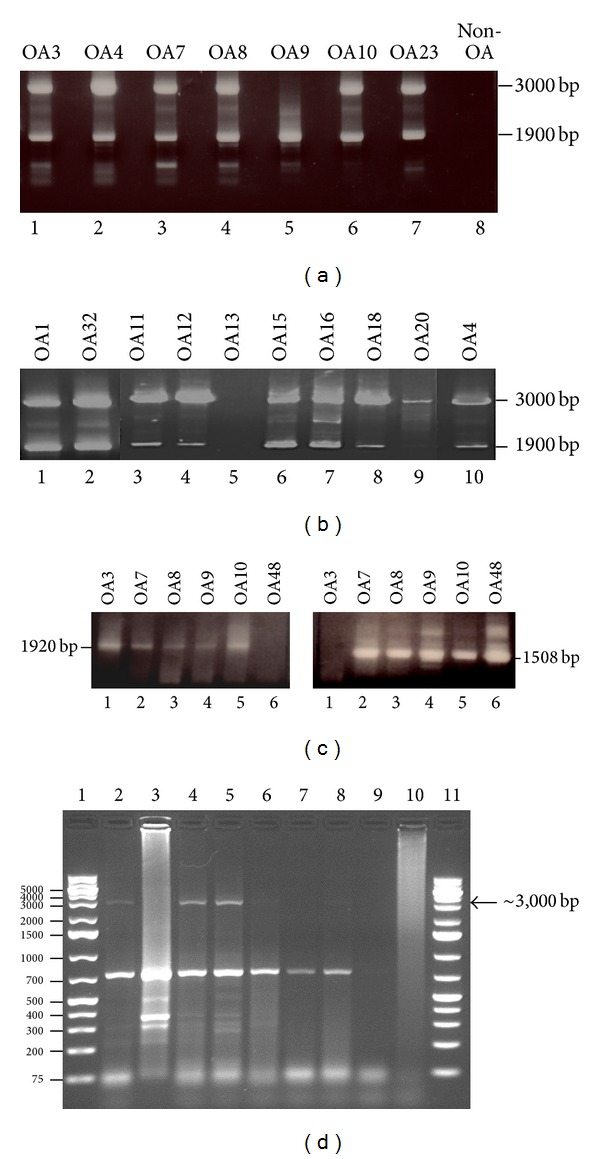
Expression of ERVWE1 and ERVW2 in chondrocytes from OA and non- or early OA patients. (a) PCR on cDNA prepared from cultured chondrocytes isolated from knees or hip from OA patients. Specific primers for the ERVWE1 and ERVWE2* env* gene (encoding syncytin) were used. (b) RNA purified from nitrogen-crushed cartilage. (c) RNA was isolated from chondrocytes obtained from 6 OA patients, converted into cDNA, and amplified using specific primers complementary to the* gag* gene of HERVWE1 (left panel) and HERVWE2 (right panel) sequences. (d) Amplification of cDNA prepared from chondrocytes from non- and early OA patients. Lanes 1 and 11: 1 kb plus ladder; lanes 2–10: amplified cDNA. The arrow indicates the presence of the amplified 3,000 bp of the ERVWE1* env* transcript.

**Figure 3:**
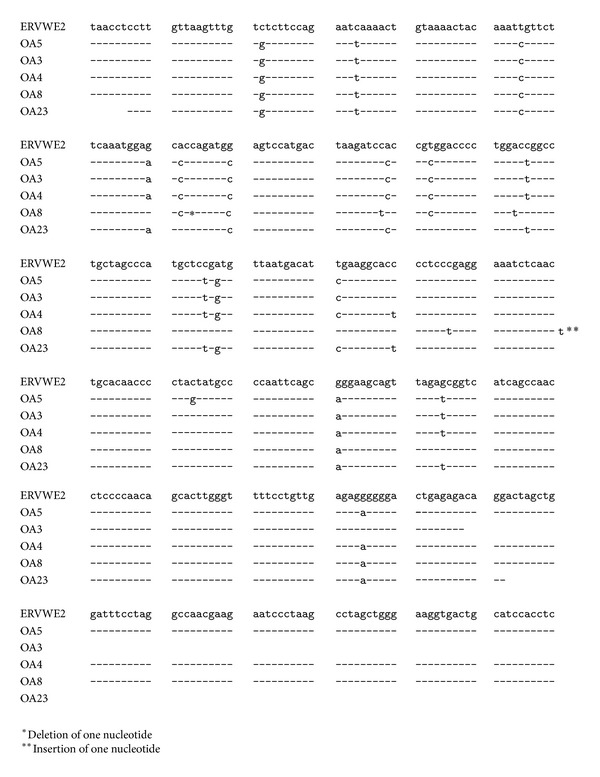
Alignment of the sequence of the 1,600 bp PCR products with ERVWE2* env* transcript. Identical nucleotides are indicated by a vertical line, while point mutations are shown. The sequence obtained from OA8 patient has a deletion and insertion which restores the ORF.

Nested PCR on the PCR reaction with inward HERV-5 primers (Table 1) did not generate PCR products. We therefore sequenced of the PCR fragments obtained with the outward HERV-5 primers. Sequence analysis revealed that DNA was identical with human endogenous retrovirus W family. Mixed PCR on cDNA was repeated with specific primers for the ERVWE1 (ERVW-1; GenBank accession number NM_014590; [33]) and the ERVWE2 (GenBank accession numbers AF127228 and AF127229; [34]) envelope genes (*env*; also referred to as syncytin). Two distinct PCR products of, respectively, ~3,000 and ~1,900 bp were detected in cartilage specimens of 15 out of 17 OA patients, but not in one non-OA patient (Figures 2(a) and 2(b); Table 2). The length of PCR products corresponds well with the theoretical length of 3013 bp and 1932 bp, respectively. Partial sequencing of the 3,000 bp PCR products confirmed that the amplified fragment spans a region of the envelope (*env*) gene encoding syncytin of the ERVW member E1 (ERVWE1; Supplementary Figure S1 [33, 35]), while the sequence data of the ~1,900 bp fragment showed >95% identity with the* env* gene of ERVWE2 (Figure 3). The viral sequences detected in our OA patients possessed point mutations compared to the reference strains ERVWE1 and ERVWE2, respectively, arguing against contamination of the samples (Figure 3 and Supplementary Figure S1). To confirm the presence of ERVW-specific transcripts in material from OA patients, PCR was performed on cDNA prepared from expanded chondrocytes from 6 OA patients (OA3, OA7, OA8, OA9, OA10, and OA48) using another primer set specific for the ERVWE1 and ERVWE2* gag* gene ([GenBank: AF156961 and AF123881], resp.; [34, 36]). ERVWE1 and ERVWE2* gag* PCR amplicons corresponding to the expected 1920 bp and 1508 bp, respectively, could be detected in the chondrocytes of all patients tested, except OA3 which had only ERVWE1* gag* transcripts and OA48 which had only ERVWE2* gag* transcripts (Figure 2(c)). No* env*-specific transcripts were detected in the chondrocytes from the non-OA patient (Figure 2(a)), but because only one non-OA patient was originally included, we expanded our control group with patients with only focal cartilage damage. We tested cDNA prepared from RNA isolated from chondrocytes from eight non-/early OA patients for the presence of ERVWE gag sequences. Weak PCR signals corresponding to the ~3,000 bp fragment were detected for 3 of these 8 patients (Figure 2(d), lanes 2, 4, and 5), while none of them were positive for the 1,600 bp fragment (Figure 2). These results suggest that ERVWE1, but not ERVWE2, may be expressed in some of the non-/early OA patients.

### 3.3. Detection of Viral dsRNA and the Viral Envelope Protein Syncytin-1

**Figure 4:**
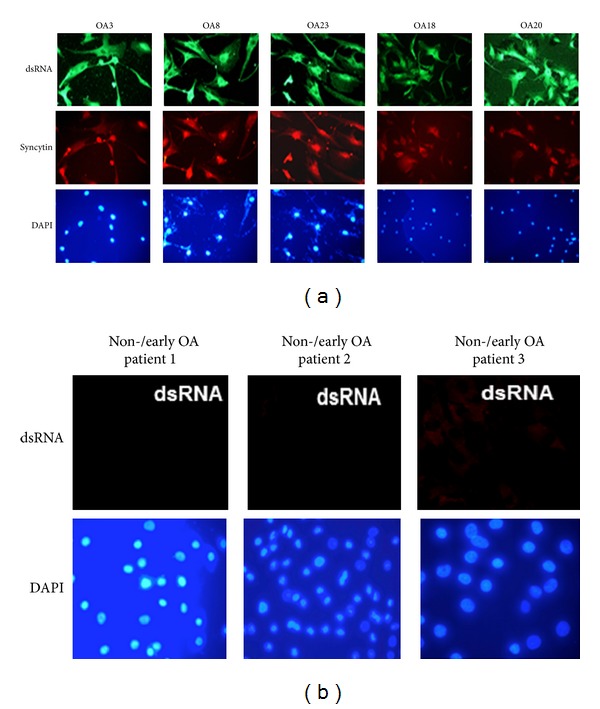
Presence of dsRNA and syncytin-1 in chondrocyte cultures obtained from OA and non- or early OA patients. Immunofluorescence assay with specific antibodies against dsRNA and syncytin was used. (a) Top row: chondrocytes from OA patients were examined for the presence of viral dsRNA by immunofluorescence using antibodies that specifically react with viral dsRNA. Middle row shows the expression of syncytin-1 in chondrocytes of the same OA patients. Bottom row: DAPI staining shows strong nuclear staining and weak cytoplasmic staining for nucleic acids. (b) Chondrocyte cultures from non-/early OA patients were monitored for the presence of dsRNA (top panel). Notice weak staining for dsRNA in chondrocytes from patient 3. Bottom row: DAPI staining.

**Figure 5:**
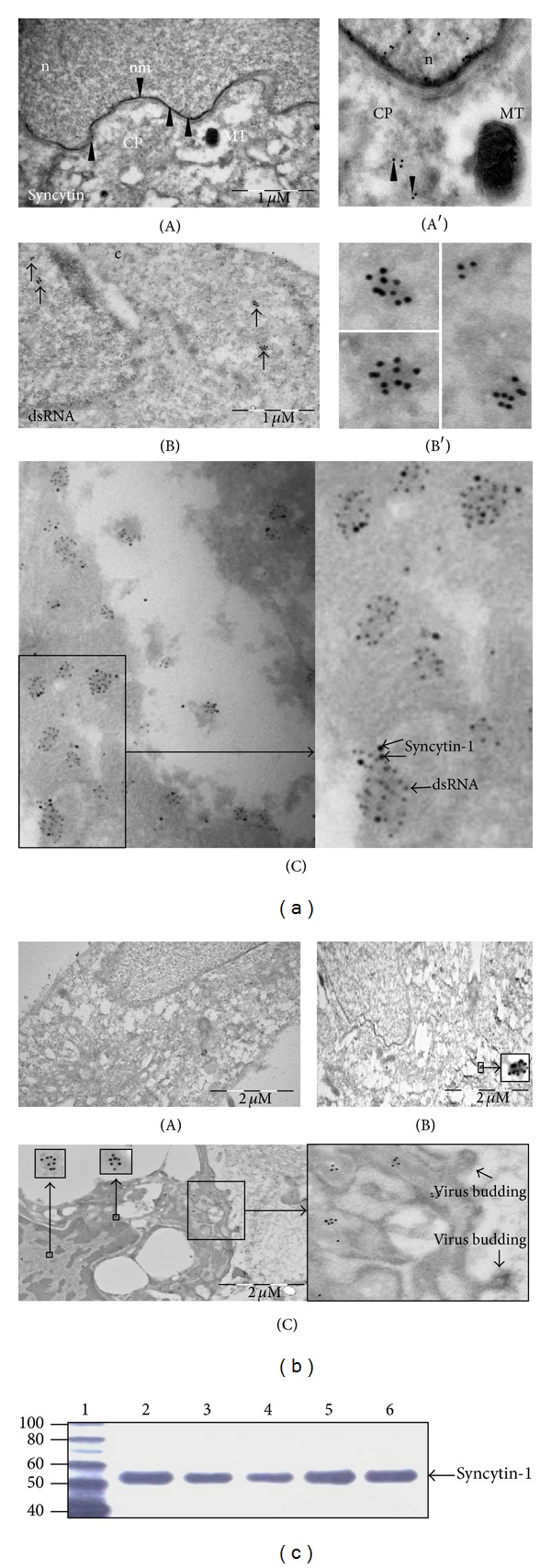
Detection of dsRNA and syncytin-1 in chondrocyte cultures obtained from OA patients by IEM. (a) IEM on samples from OA patients. Panels A and A′: cryo-IEM shows the presence of syncytin in the nucleus and cytoplasm (arrow heads). CP: cytoplasm; CM: nuclear membrane; MT: mitochondrion; n: nucleus. Panels B and B′: detection of dsRNA. Panel C illustrates that dsRNA (small dots) and syncytin (large dots) colocalize. (b) Cryo-IEM of virus dsRNA in early or non-OA patients (ACI). Panel A shows a completely negative sample and represents two out of five tested chondrocyte cultures. Panel B: example of very low levels of dsRNA detected in chondrocyte culture of a non-OA patient. Panel C: chondrocyte culture from one of the non-/early OA patients expressed relatively high levels of dsRNA. The insert depicts structures resembling virus budding (marked with an asterisk). (c) Western blot performed on lysates prepared from chondrocytes isolated from OA patients. The expression of syncytin-1 was monitored using syncytin-1 specific antibodies. A band of ~55 kDa which corresponds to the theoretic molecular mass of syncytin-1 is visible in all lysates. Lane 1: protein marker (in kDa); lanes 2–6: chondrocyte lysates.

Retroviruses pack their two copies of their genomes as dimers, and viral dsRNA can be purified from cells infected with retroviruses. Moreover, the viral genome can form long hairpin ds RNA region, for example, with its tRNA primer [37–43]. We reasoned that activation of ERVWE in chondrocytes would result in the presence of viral dsRNA. We therefore examined chondrocytes from OA and non- or early OA patients for the presence of dsRNA intermediates with an antibody that specifically recognizes longer stretches of dsRNA. This antibody will react with viral dsRNA, but not with cellular dsRNA [44, 45]. In immunofluorescence (IF) assays, chondrocytes prepared from OA cartilage samples showed a strong immunoreaction for dsRNA at both the cytoplasmic and the nuclear regions (Figure 4(a)). Additionally, traces of cytoplasmic nucleic acids could be observed in cells by DAPI staining, most likely corresponding to viral dsRNA. Chondrocytes prepared from autologous chondrocyte implantation (ACI) operations displayed no or very low (e.g., patient 3 in Figure 4(b)) staining with dsRNA-specific antibodies (Figure 4(b); Table 2). The detection of dsRNA underscores the possible presence of endogenous retrovirus, but we cannot exclude the fact that these antibodies recognize other dsRNA from other sources like RNA interference molecules or other viruses (e.g., dsRNA reoviruses). However, cartilage forms a closed system with no supply of blood and lymph and no nerves so that the presence of exogenous viruses can be excluded. Because material from non-OA had no or weak dsRNA staining, it seems unlikely that the signals in OA material derive from the RNA interference pathway. To certify HERV-W activity, samples were analyzed for expression of the envelope protein syncytin-1. Immunofluorescence showed abundant expression of syncytin-1 in OA-derived chondrocytes distributed all over the cell, while no or low syncytin-1 staining was observed in samples from non-/early OA patients (Figure 4(a); Table 3). Of the 22 OA patients examined, 20 expressed dsRNA and syncytin in their chondrocytes (Table 3). In parallel experiments, OA cartilage specimens and cells were examined by cryoimmuno-EM with the same antibodies. This procedure revealed expression of dsRNA in small clusters distributed across the cytoplasm and the nucleus (Figure 5(a), panels A and B). Interestingly, at the ultrastructural level we observed that both dsRNA and syncytin colocalized in the same clusters, scattered throughout the cytoplasm and nucleus (Figure 5(a), panel C). Of note, expression of dsRNA was not restricted to advanced OA cases since immunolabeling was achieved in cells from ACI and non-OA patients (Figure 5(b), panels B and C, and Table 3). Chondrocyte lysates from OA patients displayed immunoreactivity against syncytin-specific antibodies, suggesting the presence of syncytin in these cells (Figure 5(c)).

### 3.4. Viral Budding and Virus-Like Particles

**Figure 6:**
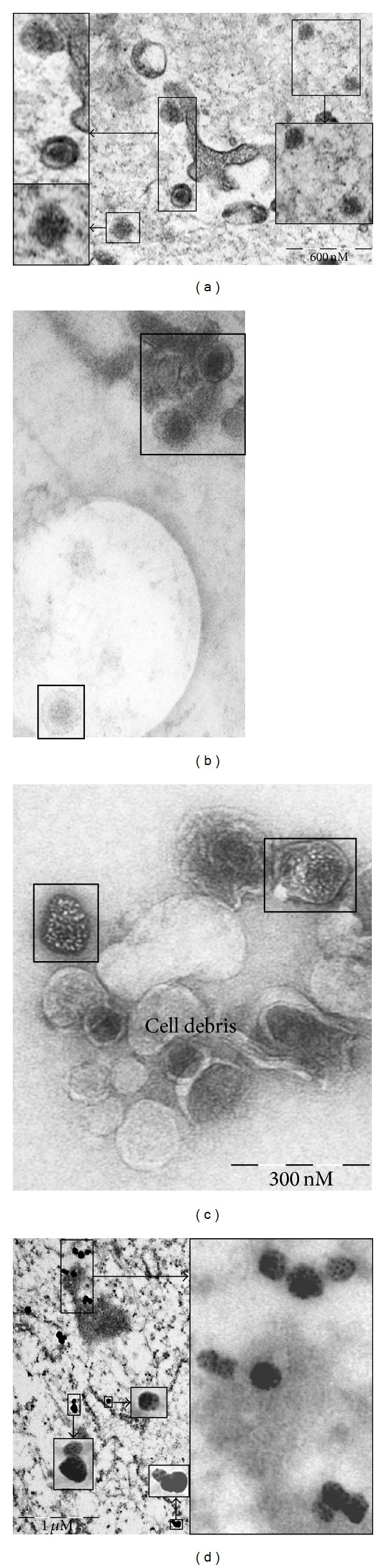
Retrovirus-like particles in cartilage of OA patients. Transmission EM (a, b, and d) and negative staining EM (c) were used to monitor the presence of virus-like particles in cartilage samples from OA patients. Negative staining EM was performed on collagenase-treated cartilage, and the resulting subcellular bodies were absorbed into grids. (d) Immune-gold labeling of viruses with antibodies against envelope protein syncytin. The size bar is indicated.

HERV genomes are integrated in the cellular chromosomes but most of these retroviral genomes are transcriptional silenced due to mutations in their coding regions or DNA methylation [46–48]. However, some HERVs can encode retroviral proteins and viral particles have been isolated [48–51]. The detection of the retroviral syncytin protein in chondrocytes and cartilage samples from OA patients prompted us to look for retrovirus particles. EM pictures taken from OA cartilage specimens showed virus-like particles holding an envelope-like structure with an estimated size of 100 nm (Figure 6), which corresponds to the size of human endogenous retrovirus particles [52–55]. Importantly, virus-like particles showed specific reactivity against syncytin-1 antibodies, as demonstrated by immune-EM gold labeling (Figure 6(d)). Similar virus-like particles also stained positively for the envelope protein syncytin in whole-mounted collagenase-digested OA-cartilage samples (Figure 6(c)). Last, we were also able to capture virus-budding processes from cell bodies in some specimens (Figure 6(a)). No retrovirus-like particles were observed in material of early or non-OA patients, except for one patient (Figure 5(b), panel C, insert). Because of the limited size of the specimens, there was not enough material to isolate and further characterize these virus particles.

## 4. Discussion

Viral infection has been suggested as an etiological factor in OA, but unequivocal proof is lacking. While viral nucleic acids and antibodies have been demonstrated in blood and synovial fluids/tissues from OA patients, studies monitoring the presence of viral activity, either as transcripts, proteins, or viral particles, in cartilage or chondrocytes from such patients have not been reported, so that a causal role of viruses in this disease remains enigmatic [6, 8, 12, 16, 18, 23, 24]. The conclusion of a contributing role of viruses in OA has also been hampered by the absent or relatively few healthy, non-OA patients examined in comparative studies. To the best of our knowledge, only one study investigated the presence of viral mRNA in cartilage of OA patients. Rollin and coworkers found HHV-4 transcripts in cartilage in 2 out of 12 OA patients, but not in twelve healthy controls [18]. We examined cartilage and cultured chondrocytes obtained from advanced OA and early/non-OA patients for the presence of viral transcripts. We could not detect mRNA from parvovirus B19, different human herpes viruses, and hepatitis C virus. However, we could show the presence of HERV activity. Our results are the first to demonstrate that activation of the endogenous retroviruses ERVWE1 and ERVWE2 occurs in cartilage/chondrocytes from OA patients and that this is a more common phenomenon in advanced than in early/non-OA patients. Activation of ERVWE1 and ERVWE2 was demonstrated here by the presence of dsRNA, ERVW-specific transcripts, expression of the viral protein syncytin, the occurrence of viral budding, and the presence of virus-like particles with morphology and size similar to the previously described MSRV and HERV-W [31, 56]. Sequencing cDNA representing part of the viral* env* transcripts revealed the presence of mutations compared to the sequences deposited in GenBank. Moreover, sequences between patients contained different mutations. This argues against contamination of our material. Several groups had detected transcripts of HERV in material of OA patients, but cartilage and chondrocytes had not been examined so far. HERV-K mRNA was identified not only in peripheral blood mononuclear cells from 17 out of 17 OA patients [19, 20] but also in synovial fluid of 4/10 (resp., 10/10, 4/4, and 2/3) OA patients [14, 17]. A recent study reported that 48% (54/113) of OA patients had detectable HERV-K18 expression in blood versus 36% (22/62) of healthy controls (*P* = 0.12), and there was an association between HERV-K18 expression and the OA severity index. These findings suggest that other HERVs may be implicated in OA [57]. Transcripts of the* polymerase* gene of HERV were detected in 3 out of 3 synovial samples from OA patients [7]. Other researchers have failed to identify HERV transcripts or retroviral particles in synovial fluid from OA patients [21, 22]. In 1999, the group of Venables reported the amplification of human retrovirus-5 (HRV-5) mRNA in synovial membranes from 3/5 OA patients, but not from control individuals (0/13), while viral DNA could be detected in synovial membranes of OA patients (3/9) and one normal subject (*n* = 29) [9, 10]. This could not be confirmed in another study, where none of the synovial tissue samples from 75 OA patients tested positive for HRV-5 DNA [26]. Later on, it was shown that HRV-5 is not integrated in human DNA and that this virus belongs to an endogenous retrovirus family found in rabbits and should be renamed RERV-H [32]. Additional pitfalls that make the interpretation of the results ambiguous are that none of the studies actually screened for viral activity in chondrocytes and that samples of control subjects (non-OA) patients often displayed similar prevalence of HERV transcript. We readily detected dsRNA and syncytin-1 in chondrocytes in 20 out of 22 tested OA patients, while weak expression levels of dsRNA and syncytin were detected in cells derived from 3 out of 7 non-OA patients that were examined. One patient (a young, healthy woman in her twenties with a sports injury, but with no sign of osteoarthritis) had high levels of both dsRNA and syncytin, and virus-like particles were present in cartilage from this patient. Although we did not use quantitative methods, fluorescence staining for dsRNA and syncytin-1 was visually weaker in non-OA patients compared to OA patients. We readily detected the presence of retrovirus-like particles in OA patients, but no such particles were observed in the cartilage biopsies taken from non-/early OA patients, except for one individual. We cannot rule out the fact that HERV particles were present in cartilage samples of chondrocytes from the other non-OA patients we examined because only a limited fraction of this material was examined. However the size of sample from non-/early OA patients was similar to that of OA patients. Our findings suggest that viral activation is at least more common in these patients compared to the control group. The observation that none of the chondrocytes isolated from non-/early OA patients (*n* = 8) possessed ERVWE2 transcripts, while 20 ERVWE2-specific transcripts were observed in 20/22 of the OA patients, may indicate that activation of this member of HERV is a hallmark for OA and can be used as a diagnostic marker.

The much higher incidence of ERVWE1/2 activation in OA patients compared to the control group may suggest that the disease status may trigger endogenous retroviral activation. Steroid hormones and inflammation are known risk factors for OA [58–63]. Interestingly, these conditions can also induce activation of HERV [64–67]. A drawback of our study is that relatively few non-OA patients were examined and their average age was significantly younger than the OA patients (range 25–45 years versus 30–82 years). For obvious reasons, it is difficult to obtain material from age-matched healthy individuals. Moreover, most samples are of very limited size making it impossible to test them by all techniques applied here in this study. The control group included younger patients suffering local cartilage damage or traumatic joint lesion (sport injuries). The increased prevalence of HERV-W in OA patients compared to the control group could therefore be age related. However, no age-related expression of HERV-W has been reported so far in other diseases where this virus has been found to be expressed. Indeed, significantly increased HERV-W* pol* transcript levels were monitored in cerebrospinal fluids from Creutzfeldt-Jakob disease patients compared to normal controls (86/87 versus 33/40; *P* = 0.001), but no correlation with sex and age existed [68]. Similarly, no significant correlation emerged between the expression of HERV-W* env* gene and age in PBMC either from autistic spectrum disorder patients or from healthy individuals [69]. The lack of a correlation between HERV-W expression and age has been reported by other groups [70, 71]. Therefore, it is unlikely that old age causes retroviral activation in cartilage of OA patients.

Assuming the implication of ERVW in the onset or progression of OA, a pivotal question that must be solved is the mechanisms by which this retrovirus contributes to the cytotoxic processes in OA. Active viral replication may contribute to the sustained inflammation of the synovial tissue that is often seen in the context of OA [59, 61]. We observe syncytin-1 expression in chondrocytes from OA patients and to a lesser extent in cells from some non-/early OA controls. Syncytin-1 is primarily produced by placental trophoblasts where it participates in cell-to-cell fusion [72, 73], but it can be expressed by some normal somatic cells. In diseased nonplacental tissue, malignant cells, as well in connection with different autoimmune diseases syncytin-1 expression has been also observed [74–79]. The role of syncytin-1 in cancer is incompletely understood, but it has been suggested to promote tumor-tumor and tumor-host cell fusion. Other groups propose a connection between syncytin expression and cell proliferation [80, 81]. Syncytin-1 has been shown to regulate inflammation in neural cells [82]. Thus, ectopic expression of syncytin-1 in human fetal astrocytes induces expression of the endoplasmic reticulum stress genes* BiP* and* XBP-1/s*, the proinflammatory cytokine* interferon α* gene, and the* NOS2* gene. In addition, syncytin-1 provokes increase in intracellular Ca^2+^ levels and supernatants from cell expressing syncytin-1 caused cytotoxic effects on oligodendrocytes [82, 83]. Intriguingly, Ca^2+^ has been shown to stimulate the activity of the enzymes matrix metalloproteases, phospholipase A2, and calmodulin-dependent kinase II, all of which may be important in the pathophysiology of OA [84–87].

## 5. Conclusions

We have shown higher prevalence of ERVWE1 and ERVWE2 activity in chondrocytes and cartilage of OA patients compared to non-/early OA patients. However, we cannot conclude whether these viruses are innocent bystanders that are activated by pathological processes occurring during the development of OA or whether they are involved in the onset or the progression/deterioration of the disease. The high prevalence of activated ERVWEs, especially ERVWE2, may be an indicator of OA, making the detection of ERVWE transcripts a putative diagnostic marker.

## Supplementary Material

The supporting data includes sequence homology between the outward HERV-5 primers and the HERV-W genome (Supplementary Figure 1) and sequence alignment of ERVWE1 gag sequences found in samples from patients with the published ERVWE1 gag sequence (GenBank accession number NM_014590; Supplementary Figure 2). The age, gender and Kellgren-Lawrence (KL) grade of the subjects participating in this study is provided in Supplementary Table 1.

## Figures and Tables

**Table 1:** Virus-specific primers used in this study.

Virus	GenBank acc. number	Primer sequences	Position
HERVWE1			
Envelope (=syncytin)	NM_014590	F: cat cga tag cac cca tca gat g	13–34
		R: gag tga aat agc atg aaa aca g	3025–3004
ERVWE1 gag	AF156961	F: tgt ccg ctg tgc tcc tga tc	1–20
		R: ctg cgc cag tgt cca gga gac	1921–1901

HERVWE2			
Envelope	AF127228,	F: cca ata gcc aga cca tta tat ac	1–23
	AF128229,	R: tgg ggt tcc att tgt aag acc	1932–1912
ERVWE2 gag	AF123881	F: cta gaa cgt att ctg gag aat tg	2–24
		R: ggc tct caa tgg tca aac ata c	1509–1488

HHV-1	GU734772	F: tcc cca taa act ggg agt agc	55192–55212
		R: cag aac tac agc gag ggc atc	55327–55307

HHV-6	NC_000898	F: gag tcc atg agt tag aag att	150874–150894
		R: cta aat ttt cta cct ccg aaa tgt	152099–152076

HHV-4 (Epstein Barr)	V01555	External primers	
		F: agg gat gcc tgg aca caa ga	48810–48829
		R: tgg tgc tgc tgg tgg tgg caa t	49406–49385
		Nested primers	
		F: tcttgatag gga tcc gct agg ata	48839–48862
		R: acc gtg gttbctg gac tat ctg gat	49335–49311

HHV-5 (human cytomegalovirus)	GQ222016	External primers	
		F: cag cac cat cct cct ctt cct ctg	3665–3688
		R: cca agc ggc ctc tga taa cca agc	4099–4076
		Nested primers	
		F: aga cac tgg ctc aga cct gac	3708–3728
		R: aga gtc tgc tct cct agt gtg	3989–3969

Hepatitis C virus	AB691598	External primers	
		F: ggc gac act cca cca tgg atc ac	16–38
		R: cat gtt gca cgg tct acg aga cc	342–320
		Nested primers	
		F: ctg tga gga act tct gtc tt	43–62
		R: ctc gca agc acc cta tca gg	309–290

Human parvovirus B19	NC_000883	External primers	
		F: aat aca ctg tgg ttt tat ggg ccg	1579–1602
		R: cca ttg ctg gtt ata acc aca ggt	1862–1839
		Nested primers	
		F: aat gaa aac ttt cca ttt aat gat gta g	1678–1705
		R: cta aaa tgg ctt ttg cag ctt cta c	1780–1756

Rabbit endogenous retrovirus (HRV5)	AF480924	External primers	
		F: tca ggt gct tca ttg gca gga tca	3008–3031
		R: taa aat ttg tac ttt tgg gca ctg ctg	3805–3782
		Nested primers	
		F: tgc aac ctt atg tta gtg cac tcc	3052–3075
		R: tac tgc ctg gtc aac ata tag	3766–3746

**Table 2:** PCR results using HERV degenerated and ERVWE1- and ERVW2-specific primers.

Patient group	ERVWE1 env (3000 bp)	ERVWE2 env (1600 bp)	ERVWE1 gag (1900 bp)	ERVWE2 gag (1500 bp)
OA	15/17 (88%)	15/17 (88%)	5/6 (83%)	5/6 (83%)
Non-/early OA	3/8 (38%)	0/8 (0%)	NT∗	NT

*NT: not tested.

**Table 3:** Prevalence of dsRNA, syncytin-1, and HERV-like particles in samples from OA and non-/early OA patients.

Material/method	IF syncytin	IF dsRNA	IEM syncytin	IEM dsRNA	TEM VLP	TEM budding
Chondrocytes OA	20/22∗ (S)∗∗	20/22 (S)	7/7 (S)	7/7 (S)	6/7	5/7
Cartilage OA			7/7 (S)	7/7 (S)	22/24	16/24
Chondrocytes non-OA	4/7 (3 W, 1 S)	3/7 (2 W, 1 S)	4/7 (3 W, 1 S)	3/7 (2 W, 1 S)		1/8
Cartilage non-OA			3/5 (2 W, 1 S)	3/5 (2 W, 1 S)		
Collagenase-treated supernatants OA					3/4	

*Number of positive samples/total number of samples.

∗∗S: strong staining; W: weak staining; VLP: virus-like particles.

## Abbreviations

ACI: Autologous chondrocyte implantation
bp: Base pairs
cDNA: Complementary DNA
dsRNA: Double-stranded RNA
EBV: Epstein Barr virus
HCV: Hepatitis C virus
HERV: Human endogenous retrovirus
HHV: Human herpes virus
HSV: Herpes simplex virus
IEM: Immunoelectron microscopy
MSRV: Multiple sclerosis-associated retrovirus
OA: Osteoarthritis
ORF: Open reading frame
PCR: Polymerase chain reaction
RT-PCR: Reverse-transcriptase PCR
TEM: Transmission electron microscopy.
