# Supplementary material for: Human Endogenous Retrovirus W Activity in Cartilage of Osteoarthritis Patients
Source: Biomed Res Int. 2014 Jul 22;2014:698609. doi: 10.1155/2014/698609 (PMC4130134; doi:10.1155/2014/698609)
Supplement: Supplementary file 1 — The supporting data includes sequence homology between the outward HERV-5 primers and the HERV-W genome (Supplementary Figure 1) and sequence alignment of ERVWE1 gag sequences found in samples from patients with the published ERVWE1 gag sequence (GenBank accession number NM_014590; Supplementary Figure 2). The age, gender and Kellgren-Lawrence (KL) grade of the subjects participating in this study is provided in Supplementary Table 1. [file 698609.f1.pdf]

Supplementary Figure 1. Sequence homology between the outward HERV-5 primers and the HERV-W genome.

```
Forward HERV-5 primer:          TCAGGTGCTT-CATTGGCAGGATCA
HERV-W:                         CTAGGTACTGGCATTGGCGGTATCA
                                  **** **  ******* * ****

Revers HERV-5 primer:      CAGCAG-----------------------------TGCCCAAAAGTAC--AAATTTTA
HERV-W:                    CAGCAGGAAGCAGTTAGAGCGGTCGTCGGCCAACCTCCCCAACAGCACTTAGGTTTTC
                           ******                             * ***** ** **  *  ****
```

Supplementary Figure 2. Sequence alignment with ERVWE1 envelope glycoprotein gene sequences 2023-2162 (NM_014590).

```
ERVWE1   CCTCCTGCTG TGCTCTCAGG CAATAGATGA TTGGCTATTT CTTTACCTCC TGTCTTTGCC
OA5      ---------- ---------- ---------- ---------- ---------- ----------
OA7                                               - ---------- ----------
OA8                                               - ---------- ----------
OA10     ---------- ---------- ---------- ---------- ---------- ----------
OA23     ---------- ---------- ---------- ---------- ---------- ----------

ERVWE1   TAATTAGCAT TTTAGTGAGC TCTCTGATTG GTCAGGTGTG AGCTAAGTTG CAAGCCCCGT
OA5      ---------- -------N-- ---------- ---------- ---------- ----------
OA7      ---------- ---------- ---------- ---------- ---------- ----------
OA8      ---------- ---------GC---------- ---------- ---------- ----------
OA10     ---------- ---------- ---------- ---------- ---------- ----------
OA23     ---------- ---------- ---------- ---------- ---------- ----------

ERVWE1   GTTTAAAGGT GGATGTGGTC ACCTTCCCAG CTAGGCTTAG GGATTCTTAG TCAGCCTAGG
OA5      ---------- ---------- ---------- ---------- ---------- ----------
OA7      ---------- ---------- ---------- ---------- ---------- ----------
OA8      ---------- ---------- ---------- ---------- ---------- ----------
OA10     ---------- ---------- ---------- ---------- ---------- ----------
OA23     ---------- ---------- ---------- ---------- ---------- ----------

ERVWE1   AAATCCAGCT AGTCCTGTCT CTCAGTCCCC CATCTCAACA GGAAAACCTA AGTGCTGTTG
OA5      ---------- ---------- ---------- ---------- ---------- ----------
OA7      ---------- ---------- ---------- ---------- ---------- ----------
OA8      ---------- ---------- ---------- ---------- ---------- ----------
OA10     ---------- ---------- ---------- ---------- ---------- ----------
OA23     ---------- ---------- ---------- ---------- ---------- ----------

ERVWE1   GGGAGGTTGG CCGACGACCG CTCTAACTGC TTCCTGCTGA ATTGGGGCGT AGTAGAGGTT
OA5      ---------- ---------- ---------- ---------- ---------- ----------
OA7      ---------- ---------- ---------- ---------- ---------- ----------
OA8      ---------- ---------- ---------- ---------- ---------- ----------
OA10     ---------- ---------- ---------- ---------- ---------- ----------
OA23     ---------- ---------- ---------- ---------- ---------- ----------

ERVWE1   GTGCAGCTGA GATTTCCTCA GGAGGGGTGC CTTTGATGTC ATTAACATCA GATCGTGGGC
OA5      ---------- ---------- ---------- ---------- ---------- ----------
OA7      ---------- ---------- ---------- ---------- ---------- ----------
OA8      ---------- ---------- ---------- ---------- ---------- ----------
OA10     ---------- ---------- ---------- ---------- ---------- ----------
OA23     ---------- ---------- ---------- ---------- ---------- ----------

ERVWE1   TAGCAGGCCG GTCCAGGGGT CTGCGGTAGA TCTTAGTCTT GGACTGCATC TTGGGCTCCA
OA5      ---------- ---------- ---------- ---------- ---------- ----------
OA7      ---------- ---------- ---------- ---------- ---------- ----------
OA8      ---------- ---------- ---------- ---------- ---------- ----------
OA10     ---------- ---------- ---------- ---------- ---------- ----------
OA23     ---------- ---------- ---------- ---------- ---------- ----------

ERVWE1   TTTGTAGTTT TACAGCTTCG ATTCTGGAAG AGACAAAGTT AACAAGGAGG TTAAAGATAC
OA5      ---------- ---------- ---------- ---------- ---------- ----------
OA7      ---------- ---------- ---------- ---------- ---------- ----------
OA8      ---------- ---------- ---------- ---------- ---------- ----------
OA10     ---------- ---------- ---------- ---------- ---------- ----------
OA23     ---------- ---------- ---------- ---------- ---------- ----------
```

```
ERVWE1   AGGGTCCAAA GAGGAGTAGC AATATTATAG CTGCTAGAGG TCCTAAGAAG GGGAGAATCC
OA5      ---------- ---------- ---------- ---------- ---------- ----------
OA7      ---------- ---------- ---------- ---------- ---------- ----------
OA8      ---------- ---------- ---------- ---------- ---------- ----------
OA10     ---------- ---------- ---------- ---------- ---------- ----------
OA23     ---------- ---------- ---------- ---------- ---------- ----------

ERVWE1   AGGGCATCCA TTGGCTGAGG AGGCCCCAGG GTCCAGTGTT TCGAAGCTCC TCTGCTCTAC
OA5      ---------- ---------- ---------- ---------- ---------- ----------
OA7      ---------- ---------- ---------- ---------- ---------- ----------
OA8      ---------- ---------- ---------- ---------- ---------- ----------
OA10     ---------- ---------- ---------- ---------- ---------- ----------
OA23     ---------- ---------- ---------- ---------- ---------- ----------

ERVWE1   GTTGTATTCG ATCTCGAATT TCTTTAACTT TCTCAGTGAC GATTCCGGAT TGATTAACAT
OA5      ---------- ---------- ---------- ---------- ---------- ----------
OA7      ---------- ---------- ---------- ---------- ---------- ----------
OA8      ---------- ---------- ---------- ---------- ---------- ----------
OA10     ---------- ---------- ---------- ---------- ---------- ----------
OA23     ---------- ---------- ---------- ---------- ---------- ----------

ERVWE1   AATAACAGCA TTCTTCCCCT AAAAATAAAC AGGTTCCCCC TCTTTCAGCG GTTAGCAAGT
OA5      ---------- ---------- ---------- ---------- ---------- ----------
OA7      ---------- ---------- ---------- ---------- ---------- ----------
OA8      ---------- ---------- ---------- ---------- ---------- ----------
OA10     ---------- ------A--- ---------- ---------- ---------- ----------
OA23     ---------- ------A--- ---------- ---------- ---------- ----------

ERVWE1   CTAAAGCTCT TCGATTTTGA AGGACTACTG CTGCTAGGGA GTTAAGTTGA TCTTGCAAGG
OA5      ---------- ---------- ---------- ---------- ---------- ----------
OA7      ---------- ----NNNN-- ---------- ---------- ---------- ----------
OA8      ---------- ---------- ---------- ---------- ---------- ----------
OA10     ---------- ---------- ---------- ---------- ---------- ----------
OA23     ---------- ---------- ---------- ---------- ---------- ----------

ERVWE1   TGACCAGGGA GTCGGCGACC CGTTCCATGT CCCCATTTAG TTCTTGAGAT AGTTTGTAGT
OA5      ---------- ---------- ---------- ---------- ---------- ----------
OA7      ---------- ---------- ---------- ---------- ---------- ----------
OA8      ---------- ---------- ---------- ---------- ---------- ----------
OA10     ---------- ---------- ---------- ---------- ---------- ----------
OA23     ---------- ---------- ---------- ---------- ---------- ----------

ERVWE1   AGAACTGAGT AGAGGTTGTG ATACCGCCAA TGCCAGTACC TAGTGCACCT AGCACTCCTG
OA5      ---------- ---------- ---------- ---------- ---------- ----------
OA7      ---------- ---------- ---------- ---------- ---------- ----------
OA8      ---------- ---------- ---------- ---------- ---------- ----------
OA10
OA23
```

Supplementary Table.

Patient	year of birth	Age at sampling	KL grade
OA1	missing		
OA2	1947	59	2
OA3	1960	46	2
OA04	1932	74	1
OA5	1965	41	3
OA7	1929	77	1
OA8	1937	69	1
OA9	NA		
OA10	1945	61	2
OA11	1970	36	3
OA13	1938	68	1
OA14	1940	66	1
OA15	1939	67	1
OA16	1950	56	2
OA17	1944	62	2
OA18	1959	47	2
OA12	1970	36	3
OA20	NA		
OA21	1971	35	3
OA22	1929	77	1
OA23	1929	78	1
OA24	1944	63	2
OA25	1940	67	1
OA26	1951	56	2
OA27	1939	68	1
OA28	1925	82	1
OA29	1934	73	1
OA30	1930	77	1
OA32	1982	25	3
OA40	1970	37	3
OA44	1944	63	2
OA48	1927	80	1
OA50	1952	56	2
OA51	1950	58	2
OA58	1929	79	1

NA: not available.
